# Brazilian Guidelines for Nutrition in Cystic Fibrosis

**DOI:** 10.31744/einstein_journal/2022RW5686

**Published:** 2022-03-22

**Authors:** Lenycia de Cassya Lopes Neri, Miriam Isabel Souza dos Santos Simon, Valéria Laguna Salomão Ambrósio, Eliana Barbosa, Monique Ferreira Garcia, Juliana Ferreira Mauri, Renata Rodrigues Guirau, Mirella Aparecida Neves, Carolina de Azevedo Pedrosa Cunha, Marcelo Coelho Nogueira, Anna Carolina Di Creddo Alves, Jocemara Gurmini, Maria de Fatima Servidoni, Matias Epifanio, Rodrigo Athanazio

**Affiliations:** 1 Hospital das Clínicas Faculdade de Medicina Universidade de São Paulo São Paulo SP Brazil Instituto da Criança, Hospital das Clínicas, Faculdade de Medicina, Universidade de São Paulo, São Paulo, SP, Brazil.; 2 Universidade Federal do Rio Grande do Sul Porto Alegre RS Brazil Universidade Federal do Rio Grande do Sul, Porto Alegre, RS, Brazil.; 3 Hospital das Clínicas Faculdade de Medicina de Ribeirão Preto Universidade de São Paulo Ribeirão Preto SP Brazil Hospital das Clínicas, Faculdade de Medicina de Ribeirão Preto, Universidade de São Paulo, Ribeirão Preto, SP, Brazil.; 4 Hospital Infantil Joana de Gusmão Florianópolis SC Brazil Hospital Infantil Joana de Gusmão, Florianópolis, SC, Brazil.; 5 Escola Paulista de Medicina Universidade Federal de São Paulo São Paulo SP Brazil Escola Paulista de Medicina, Universidade Federal de São Paulo, São Paulo, SP, Brazil.; 6 Universidade Estadual de Campinas Campinas SP Brazil Universidade Estadual de Campinas, Campinas, SP, Brazil.; 7 Hospital Pequeno Príncipe Curitiba PR Brazil Hospital Pequeno Príncipe, Curitiba, PR, Brazil.; 8 Hospital da Criança de Brasília José Alencar Brasília DF Brazil Hospital da Criança de Brasília José Alencar, Brasília, DF, Brazil.; 9 Hospital Infantil João Paulo II Belo Horizonte MG Brazil Hospital Infantil João Paulo II, Belo Horizonte, MG, Brazil.; 10 Hospital das Clínicas Faculdade de Medicina Universidade de São Paulo São Paulo SP Brazil Instituto do Coração (InCor), Hospital das Clínicas, Faculdade de Medicina, Universidade de São Paulo, São Paulo, SP, Brazil.; 11 Pontifícia Universidade Católica do Rio Grande do Sul Porto Alegre RS Brazil Pontifícia Universidade Católica do Rio Grande do Sul, Porto Alegre, RS, Brazil.

**Keywords:** Nutrition therapy, Cystic fibrosis, Nutritional assessment, Recommended dietary allowances

## Abstract

**Objective:**

To develop a scientific consensus on nutrition in cystic fibrosis.

**Methods:**

Sixteen coordinators elaborated relevant questions on nutritional therapy in cystic fibrosis, which were divided into six sections: nutritional assessment, nutritional recommendations, nutritional intervention, dietary counseling, special situations and enzyme replacement, and gastrointestinal manifestations. Two to three specialists in the field were responsible for each section and obtaining answers formulated based on standardized bibliographic searches. The available literature was searched in the PubMed^®^/MEDLINE database, after training and standardization of search strategies, to write the best level of evidence for the questions elaborated. Issues related to disagreement were discussed until a consensus was reached among specialists, based on the current scientific literature.

**Results:**

Forty-two questions were prepared and objectively answered, resulting in a consensus of nutritional therapy in cystic fibrosis.

**Conclusion:**

This work enabled establishing a scientific consensus for nutritional treatment of cystic fibrosis patients.

## INTRODUCTION

Cystic fibrosis (CF) is an autosomal recessive inherited lethal disease, with over two thousand different mutations on chromosome 7, which encodes the cystic fibrosis transmembrane conductance regulator (CFTR) protein, and mainly causes alterations in the transport of ions and water in cells of the respiratory, gastrointestinal, hepatobiliary and reproductive systems, as well as in sweat glands.^(1)^

There is a strong association between lung function and nutritional status, so that nutritional aspects should be monitored throughout treatment of these patients.^(2)^

The importance of nutritional intervention in CF patients has been highlighted since the landmark publication by Corey et al., who compared two CF treatment clinics in Toronto and Boston, in which patient survival was different, favoring the center with nutritional therapy that offered a high-fat diet.^(3)^ Nutritional aspects of CF have been the subject of several international publications, and many expert committees from various countries have described nutritional guidelines focused on different age groups.^(1,4-9)^

In 2016, the European Society for Clinical Nutrition and Metabolism/European Society for Paediatric Gastroenterology, Hepatology and Nutrition (ESPEN/ESPGHAN), together with the European Cystic Fibrosis Society (ECFS), published an important guideline with nutritional recommendations for infants, children, adolescents, and adults with CF.^(4)^ This guideline included evidence-level orientation on nutritional assessment; macro- and micronutrient recommendations; management of pancreatic insufficiency and malnutrition; oral, enteral, and parenteral nutritional intervention; behavioral intervention; and management of CF-related diabetes, gastrointestinal complications, bone disease, liver disease, and specialized nutrition, with great value for the clinical nutritional management of these patients.^(4)^ In any case, there was a need for a national document to gather recommendations for nutritional treatment based on the realities of our country.

Since there are no guidelines or national consensus on nutrition in CF, this document was developed in partnership with experts from reference centers of all Brazilian regions, to answer the main questions related to nutrition management in CF.

## OBJECTIVE

To develop a scientific consensus on nutrition in cystic fibrosis.

## METHODS

A group of 16 professionals directly involved in CF nutritional care, from several reference centers in Brazil, coordinated this guideline. Participants included dietitian, gastroenterologists, physician nutrition specialists, and pulmonologists. These coordinators were responsible for the following tasks: preparing relevant questions to be answered in their respective sections; inviting authors working in the field and with expertise to prepare answers to their questions and revise the content of the answers in their section. One of these professionals was also chosen as the general coordinator and had the following attributions: distribute the coordinators among the consensus sections according to their respective areas of expertise; set deadlines and distribute tasks to all coordinators; follow the process of developing the answers; discuss divergent issues with coordinators until consensus was reached; and compile, revise, and produce a single, coherent, and clear document.

This team developed a list of topics relevant to the nutritional care of the CF patient, separated into six sections: nutritional assessment, nutritional recommendations, nutritional intervention, dietary counseling, special situations and enzyme replacement, and gastrointestinal manifestations.

The questions were adapted in their format to favor objective and practical answers. Those with no relevance to CF treatment and duplicates were eliminated.

The group conducted an online training session to standardize searches, covering topics such as selection of keywords using the PICO (population, intervention, control, and outcome) strategy; verification of standardized descriptors for the search; how to search databases, with standardization of the PubMed^®^/MEDLINE database for literature searches; and how to choose articles to support the answers to the questions, to obtain the best level of evidence. The Oxford Centre for Evidence-Based Medicine (CEBM) model was adopted to classify the questions. This model was based on the same methodology applied to the Brazilian cystic fibrosis guidelines.^(10)^

The first version was written using files shared online among coordinators, between July 2017 and September 2018. After this period, a face-to-face meeting was held with all coordinators to discuss, standardize, and finalize the answers. At that time, other questions, which did not fit the nutritional aspects *per se*, were eliminated, leaving only 42 questions in this consensus. Some differences of opinion were also discussed and standardized.

The final version was subjected to a standardization of the level of evidence for each question, according to the types of articles used in the references. When the document was finalized by all section coordinators, the consensus was reviewed by two coordinators.

## RESULTS

The following are the six sections, with their respective questions and answers, classified by level of scientific evidence.

### 1. Nutritional assessment

#### Is the nutritional status of cystic fibrosis patients related to their prognosis?

Yes. The nutritional status has an important relation with the long-term evolution of lung disease, since it is related to the quality of life and survival of these patients.^(2,11,12)^ Children and adults with better body mass index (BMI)^(8)^ and greater muscle mass,^(13)^ have better lung function and survival^(14)^ (level of evidence 2).

#### What parameters are used for nutritional assessment in cystic fibrosis?

Anthropometric (weight, height, and head circumference), body composition (fat mass and muscle mass), food intake, and biochemical parameters are used. The difference between parameters and life cycles refers to the anthropometric parameters. For adults, BMI in absolute value is used. For children and adolescents, percentiles and z-scores are used: BMI/age, weight/age, weight/height, and height/age.^(4)^ For infants, the head circumference is also used, evaluated at each visit^(5)^ (level of evidence 5).

#### When to monitor the nutritional status of patients with cystic fibrosis?

For breastfeeding infants, monitoring of nutritional status should be performed every 1 to 2 weeks, until evidence of adequate nutrition and ideal nutritional status is established; thereafter, it can be done monthly during the first year of life, if possible, even throughout early childhood.^(4)^ Children and adults should be monitored every 3 months.^(4)^Assessments should be more frequent when patients present with malnutrition: every 2 weeks for infants and every 6 to 8 weeks for children and adults.^(15)^ Body composition analysis and biochemical tests should be performed annually^(16)^ (level of evidence 5).

#### Can we use the same growth curves of healthy children for cystic fibrosis?

Yes. The growth curves used in Brazil and recommended for CF patients are those of the World Health Organization (WHO), 2006 and 2007^(17)^(level of evidence 5).

#### Can we use the same nutritional status classification criteria for patients with cystic fibrosis?

No. The nutritional status classification criteria are specific for CF and are described on table 1 (level of evidence 5).

**Table 1:** Classification of nutritional status according to age group(4,6,7,9,15,18,19)

Nutritional status	Infants <2 years*	Children and adolescents 2 to 18 years^†^	Adults
Optimal	W/H ≥p50 W/A ≥p50 H/A ≥p50 (z-score ≥0)	BMI p50-p85 (z-score 0 – z-score +1) 2-5 years W/H ≥p10 (z-score ≥-1.28)	Women: BMI 22-25kg/m^2^ Men: BMI 23-25kg/m^2^
Eutrophic	W/H p25-p50 (z-score 0.67 – z-score 0)	BMI p25-p50 (z-score 0.67 – z-score 0) 2-5 years W/H ≥p10 With no recent loss of weight	Women: BMI 20-22kg/m^2^ Men: BMI 20-23kg/m^2^ With no recent non-intentional weight loss
Nutritional risk	W/H p10-p25 (z-score 1.28 – z-score 0.67)	BMI p10-p25 Weight loss or plateau in 2 to 4 months	BMI 18.5-20kg/m^2^ and/or ≥5% non-intentional weight loss over the last 2 months
Malnutrition	W/H <p10 (z-score <-1.28) H/A <p5 and/or drop of 2 percentiles in growth	BMI < p10 (z-score <-1.28) and/or drop of 2 percentiles in weight and inflection of growth	BMI <18.5kg/m^2^ On-going weight loss (>5%)
Overweight		BMI p85-p97 (z-score +1 – z-score +2)	BMI 25-30kg/m^2^
Obesity		BMI >97 (>z-score +2)	BMI >30kg/m^2^

* Consider W/H preferably to BMI/A in infants; ^†^ in all nutritional classifications of children and adolescents, the H/A indicator should be considered based on genetic potential (H/A ≥genetic potential).^(4,15)^

W: weight; H: height; A: age; BMI: body mass index.

#### Can we use the same methods to assess body composition and pubertal development in cystic fibrosis patients?

Yes. To assess body composition, bone densitometry by dual-energy X-ray absorptiometry (DXA) and electrical bioimpedance are recommended whenever possible.^(7,20)^ Specifically for adult CF patients, ESPEN/ESGHAN proposes the fat-free mass index, with a cut-off point for malnutrition when <15kg/m^2^ for women and <17kg/m^2^ for men.^(4)^

For the evaluation of sexual maturation, the Tanner staging is suggested.^(21)^ However, it is important to pay attention to growth rate, which can occur at the same age, but with reduced magnitude in boys, and later and attenuated in girls, accounting for a delay or problem in growth rate^(22)^ (level of evidence 5).

#### Are there biochemical tests that can contribute to assessment of nutritional status in cystic fibrosis*?*

Yes. Tests such as complete blood count, serum iron, urea, electrolytes, fat-soluble vitamins, fatty acids, liver and kidney function, and inflammatory markers.^(4)^ However, there are no specific biochemical parameters indicating or validating the presence of malnutrition in CF.^(23)^ If the patient presents malnutrition and/or recovery of nutritional status and/or presents changes in biochemical tests, reassessment within 3 months is recommended. For patients aged 10 years or more, an oral glucose tolerance test is also recommended^(4)^ (level of evidence 5).

#### Is there relevance in the evaluation of inflammatory markers for nutritional assessment in cystic fibrosis?

Yes. Inflammatory markers, such as visceral proteins (albumin, prealbumin, transferrin, and retinol-binding protein) are important for nutritional assessment in CF. However, they should not be used alone to diagnose the nutritional status, since these proteins are affected by other clinical factors, such as cytokine release, plasma protein concentrations, and muscle catabolism^(4,24-26)^ (level of evidence 5).

#### Can we use the same dietary intake assessment methods for patients with cystic fibrosis?

Yes. The application of the 24-hour recall, the dietary record for 3 to 5 days, and the retrieval of the patient’s dietary history are recommended for a more detailed quantitative assessment of energy and nutrient intake. It should also contain questions about adherence to dietary counseling, through nutritional education of the patient and his family^(4,7,20)^ (level of evidence 5).

## 2. Nutritional recommendations

### Does the caloric demand of individuals with cystic fibrosis differ from that of the normal population?

Yes. Energy requirements are higher in CF patients, ranging from 110 to 200% of the caloric value recommended for the healthy population, considering age and sex. The variation is due to individual factors, such as current nutritional status, lung function, presence of infections, and periods of worsening general condition^(8)^ (level of evidence 2).

### Are there specific carbohydrate recommendations for cystic fibrosis?

Yes. In CF patients, carbohydrates are the main source of energy in the diet. It is recommended that 40% to 45% of daily energy intake should come from carbohydrates adjusted to caloric requirements^(1,4,8,27)^ (level of evidence 5).

### Is there a specific protein recommendation for cystic fibrosis?

Yes. Protein intake should be 20% or more of caloric intake, as in other inflammatory diseases, aiming to maintain or recover lean body mass and improve prognosis.^(1,4,8,27)^It is recommended that two-thirds of the protein intake come from high biological value protein sources.^(28)^ The ideal protein intake should be evaluated individually, since it depends on several factors, such as digestibility, efficacy of pancreatic enzymes, presence of chronic pulmonary inflammation, and disease exacerbations^(29)^ (level of evidence 5).

### Are there specific recommendations for fats in cystic fibrosis? When is it necessary to supplement essential fatty acids?

Yes. Fat intake should be higher than that recommended for the general population, accounting for 35% to 40% of energy ingestion, to increase energy intake and maintain and/or recover nutrition.^(1,4,8,27)^

Ideally, lipid sources should be distributed in approximately one-third saturated fatty acids, up to one-third polyunsaturated fatty acids, and the rest monounsaturated, but no trans fatty acids. Pediatric and adult patients should be instructed to eat foods rich in polyunsaturated and monounsaturated fats^(8,27)^ (level of evidence 5).

### Is there a specific fiber recommendation for cystic fibrosis?

There are no specific recommendations for fiber intake in CF patients. Adequate fiber intake is recommended, according to the Dietary Reference Intakes (DRIs). Even in cases of constipation, there is no solid evidence of the need to supplement this nutrient, and it is only recommended to adjust the diet with the optimal amount^(8,27,29)^ (level of evidence 5).

### Is there a need for sodium supplementation in cystic fibrosis patients?

Yes. Increased sodium chloride intake is recommended at all ages, especially in warmer periods of the year; if there is body fever; with vigorous exercise; tachypnea; fluid loss from diarrhea or vomiting or ostomy losses; if there is unexplained nutritional failure and/or urinary sodium dosage (under 24 months), and/or fractional urine sodium excretion show body sodium depletion.^(9,15,30,31)^

Neonates and infants should take 2.5-3.0mEq/kg per day; children, up to mEq/kg per day; and for adolescents and adults, increase the intake of salty foods and/or add a fluid and electrolyte replacement throughout the day, as needed (level of evidence 4).

### Is there a need for mineral (calcium, iron, zinc, and selenium) supplementation in cystic fibrosis patients?

Mineral supplementation in CF patients should occur as needed, respecting the recommendations for each mineral, according to guidelines for the general population. However, some points deserve attention regarding the treatment of CF and mineral supplementation:

- Iron: supplementation should not be performed close to the enzyme replacement time, in cases of fever or infections. In cases of iron deficiency, it is recommended to wait until treatment of the inflammation is completed, supplementing with iron only if the deficiency persists. Ferritin should not be evaluated as an isolated parameter for iron stores in cystic fibrosis patients^(9,15)^ (level of evidence 4).
- Calcium: calcium intake should be frequently evaluated, especially in children with weight and growth *deficits*, prolonged use of corticosteroids, pancreatic insufficiency, hypovitaminosis D, and daily dietary intake should be encouraged, according to recommendations ^(9,30)^ (level of evidence 5).
- Zinc: supplementation is recommended in individuals with growth failure, malnutrition, hyporexia, immune depletion, delayed sexual maturation, insufficient vitamin A levels, or night blindness that do not respond to vitamin A supplementation. It is recommended to administer zinc supplementation together with pancreatic enzymes, and in fractionated form for better absorption^(9,15)^ (level of evidence 5).
- Magnesium: replacement should only be done in patients on long-term treatment with aminoglycosides and severe malabsorption^(9)^ (level of evidence 5).
- Selenium: there is no evidence of benefit from selenium supplementation in CF patients (level of evidence 5).

### Is there a need for fat-soluble vitamin supplementation in cystic fibrosis patients? How are this supplementation and monitoring performed?

Yes. For all CF patients with pancreatic failure, for which it is recommended to monitor plasma levels of fat-soluble vitamins after the start of vitamin supplementation, 3 months after the start of supplementation, and annually. For patients with pancreatic sufficiency, monitoring should be annual, with no need for supplementation, except in specific cases of deficiency.^(8,27)^

When biochemical deficiency is detected despite adequate vitamin supplementation, poor compliance, or poor absorption of supplements should be taken into account before adjusting the dosage.

Recommendations for supplementation of fat-soluble vitamins are shown on table 2 (level of evidence 2 for vitamin E; level of evidence 5 for vitamin A; level of evidence 3 for vitamin D and level of evidence 2 for vitamin K).

**Table 2:** Fat-soluble vitamin supplementation(8,32-35)

Vitamin A	<1 year: 4,000IU
	>1 year: 4,000IU-10,000IU
	Adults: 4,000IU-10,000IU
Vitamin D	<1 year: 400IU
	>1 year: 400IU-800IU
	Adults: 800IU-2,000IU
Vitamin E	<1 year: 50IU/day
	>1 year: 100IU-400IU/day
	Adults: 100IU-400IU/day
Vitamin K	<1 year: 0.3mg-1.0mg/day
	>1 year: 1mg-10mg/day

### Is it necessary to supplement water-soluble vitamins in cystic fibrosis?

There is no evidence of the clinical efficacy of supplementation of B-complex vitamins or vitamin C in the routine treatment of CF patients, because the use of nutritional supplements, frequent in CF patients, increases the intake of these vitamins, which are better absorbed than fat-soluble vitamins^(1,8)^(level of evidence 5).

### Is antioxidant supplementation necessary?

There is no satisfactory evidence regarding the efficacy of antioxidant supplementation. Some data suggest the use of glutathione, zinc, vitamin A, vitamin C, and vitamin E may favor the reduction of oxidative stress markers. However, more studies are needed regarding the dosage and the positive effect of their use^(30,35-38)^ (level of evidence 5).

## 3. Nutritional intervention (oral supplementation, enteral and parenteral nutrition therapy)

### What is the impact of nutritional intervention in the clinical evolution of cystic fibrosis patients?

Regarding oral nutritional supplementation (ONS), in clinical practice, short-term use has been shown to increase energy intake and weight in malnourished children.^(39,40)^ However, most studies have observed that ONS does not promote additional weight gain in moderately malnourished CF children, compared with those followed only with dietary counseling.^(39,41-44)^ Nevertheless, studies using oral supplementation with high energy density showed an increase in weight^(40,45)^ and arm muscle circumference.^(45)^ Thus, due to limited evidence, the results should be interpreted with caution, because the use of ONS and its benefits should be evaluated individually^(4)^ (level of evidence 3).

When oral nutritional therapy fails, enteral nutrition therapy (ENT) is a treatment option used exclusively or partially to replace or supplement oral feeding in malnourished or non-nutritionally ill patients, according to their nutritional requirements.^(46)^

According to a Cochrane systematic review, no eligible randomized controlled trial evaluating the efficacy of ENT in CF was identified.^(47)^ However, non-randomized studies showed several beneficial effects of ENT in CF patients, such as increased energy supply,^(48)^ weight gain ^(49-57)^ and BMI.^(53-55)^ It has also been observed to increase growth rate - especially in children^(48,49,53)^ increase body fat^(49,58)^ and muscle mass, as well as improve biochemical markers.^(57,59)^ In addition, ENT has contributed to the stabilization^(48,49,51,52,54,55)^ or even improvement of lung function^(50,51,56,57)^ (level of evidence 4).

Parenteral nutrition therapy (PNT) is not routinely used as nutritional therapy in CF patients, but its use may be recommended in cases where ENT is contraindicated^(20)^ (level of evidence 5).

### When and how to implement oral nutritional supplementation in cystic fibrosis patients?

Oral nutritional supplementation is recommended when the patient presents with moderate or high nutritional risk, with reduction in percentile (p) and/or z-score for weight and height,^(60)^ and when nutritional requirements are not met with food, despite dietary counseling and adequate enzyme replacement.^(4)^ It may also be indicated in situations of respiratory aggravation or other situations whereas there is reduced food intake.^(60,61)^It is important to investigate other causes that may trigger reduced food intake and altered nutritional status, such as Giardia infection, celiac disease, hypercatabolism by lung disease, vomiting or gastroparesis, glycosuria, or CF-related diabetes, psychological impacts,^(62)^ and/or behavioral problems.^(4)^

The criteria for ONS indication are as follows: for children ≤2 years, weight and length between p10-p50^th^, or weight loss or decline in weight or length;^(4,16)^ for children between 2 and 18 years, BMI between p10-50^th^ or weight loss in the previous 2 to 4 months, or no weight gain in a 2-month period;^(4)^ and for adults, BMI 20 to <23kg/m^2^ for men, and 20 to <22kg/m^2^ for women, or weight loss of 5% in the previous 2 months.^(62)^

It is recommended that ONS be added to the diet in an equivalent amount of 20%^(41,42)^or 25%,^(43)^according to their nutritional requirements. It should be prescribed individually, taking into consideration the patient’s age range, preferences, and clinical and nutritional status.^(63)^

To ensure the ONS does not replace the usual meals and does not impair appetite, with a consequent reduction in regular food intake, it is recommended to take it between meals and before bedtime. It is important to take into account personal preferences to minimize its long-term rejection^(4,7,60)^ (level of evidence 3).

### When and how to implement enteral nutrition therapy in cystic fibrosis patients?

The implementation of nutritional therapy via nasogastric tube, nasojejunal tube, or gastrostomy should be recommended by consensus among the pulmonologist, gastroenterologist, physician nutrition specialist, nurse, and social worker.^(50,55)^ Prior psychological evaluation is important to identify psychosocial factors of the patient and family.^(64)^ Among the criteria to implement enteral nutritional therapy, one can consider continuous weight loss or inadequate weight gain, in a period of 3 to 6 months, even with the use of ONS, optimization of enzyme replacement therapy, and strict dietary counseling with regular follow-up;^(4,50,53,55,57)^ for children and adolescents with difficulty in reaching p10-25^th^ for the BMI/age indicator or weight/age or height/age <p10^th^;^(50,52,55,57)^ for adults with persistent BMI <18.5kg/m^2^;^(4,40-45)^ in cases of anorexia with reduced food intake of at least 120% of recommendation (Recommended Dietary Allowances - RDAs) for energy,^(57)^ and without medical contraindication. In case of gastrostomy, consider the pulmonary and cardiac functions of the patient.^(64)^

Enteral nutrition therapy should be individualized for the requirements of the patient and, generally, should furnish between 40% and 60% of the daily need for energy. The use of supplementary feeding administered continuously during the night is suggested, varying from three to seven times a week, maintaining oral feeding normally during the day.^(48-51,53,54,56,57,64)^ The selection of the enteral formula, made by dieticians, should include issues of composition and energy density.^(55)^Except for contraindications, the polymeric formula is recommended according to preference or availability, with energy density from 1.0kcal/mL to 1.5kcal/mL, and up to 2.6kcal/mL in adults. If necessary, carbohydrate, protein, and/or lipid modules may be used to increase the energy density.^(48-56,65)^ Enteral nutrition therapy via tube or gastrostomy should be started at the hospital, with a progressive volume increase during the first 5 days, according to tolerance.^(57,65)^ The patient should be discharged after showing tolerance to the enteral diet with recommended volume and energy intake, as well as appropriate clinical and biochemical parameters.^(65)^ It is suggested that, during hospitalization, patients and/or their families receive training for correct administration of this therapy^(57)^(level of evidence 4).

### Is there a specific indication for parenteral nutrition therapy in cystic fibrosis patients?

There is no specific indication for CF patients. The guidelines are the same as for the general population. Thus, PNT should be used in cases in which ENT is contraindicated, such as intestinal resection, short bowel syndrome, severe pancreatitis, severe gastroenteritis, and in the postoperative period, when indicated by the multidisciplinary team for the shortest possible time^(8,66-69)^ (level of evidence 5).

### What parameters should be monitored in cystic fibrosis patients with oral supplementation and enteral nutritional therapy? How often should monitoring be performed? When should nutritional intervention be suspended?

For monitoring, it is recommended to evaluate anthropometric, biochemical, food intake, and enzyme replacement parameters, in addition to clinical aspects and gastrointestinal tolerance.^(50,54,56,57)^

Regarding anthropometric parameters, change in weight and height, BMI, arm circumference, triceps skinfold thickness, and arm muscle circumference can be assessed. Electrical bioimpedance can also be used for body composition assessment.^(41-43,45,54,56,57,70,71)^ The importance of assessing body composition markers is emphasized, considering the role of lean mass in improving lung function.^(11)^

Among the biochemical parameters, evaluation of serum albumin, pre-albumin, hematocrit, hemoglobin, transferrin, retinol-binding protein, retinol, alpha-tocopherol, zinc, and/or copper, urea, electrolytes, fecal fat, and glycemia are suggested.^(41,45,50,54,56,57,71)^For food intake, a 24-hour recall, or a 3 to 5 days dietary record is recommended.^(41,42,50,54,56,57,71)^ Also, when possible, clinical scores may be calculated and changes in respiratory function (forced expiratory volume in one second - FEV_1_) and liver function may be evaluated.^(41,42,45,50,54,56,57,71)^

In patients with ONS, the parameters used for monitoring should be evaluated at baseline and sequentially, at regular intervals,^(7)^to determine the continuity of oral supplementation.^(4)^In ENT, patients should be evaluated 7 to 10 days after placement of the tube or gastrostomy, monthly for the first 3 months, and every 3 months thereafter. The frequency of evaluation may vary according to the patient’s clinical condition.^(50,54,56,57)^

Tolerance assessment must consider the patient’s sense of well-being and the presence of complications related to feeding, such as altered frequency of bowel movements, nausea, and vomiting, abdominal distension, and enzyme replacement.^(41,42,45,50,54,65,71)^

Discontinuation of the ONS may be considered when patients using it show intolerance and low compliance, or do not show improvement in nutritional status with its use,^(4,19)^ or when the child/adult reaches appropriate nutritional status (Table 1) and shows habitual food intake that meets their nutritional requirements (level of evidence 5).

### Which parameters should be monitored in cystic fibrosis patients on parenteral nutrition therapy?

The parameters are the same for any patient receiving PNT: anthropometric, laboratory, and clinical parameters. Monitoring should be strict during the first days, and a weekly control scheme can be made to facilitate and standardize the follow-up of these patients, which does not exclude an individualized assessment,^(72)^ according to table 3.

**Table 3:** Monitoring parenteral nutrition patients(72)

Data*	Start and first week (weekly frequency)	Up to 3 weeks (weekly frequency)	Up to 8 weeks (monthly frequency)	After months (monthly frequency)
Weight of infants	7	3	8	2
Weight of children	3	2	2	1
Sodium and potassium	7	3	4	2
Ionic calcium	3	3	2	1
Phosphorus	3	3	2	1
Magnesium	3	3	2	1
Complete blood count	2	1	2	1
Urea and creatinine	3	2	4	1
Blood glucose	28	14	30	1
Glycosuria	14	7	4	1
Alanine aminotransferase	1	1	2	1
Gamma glutamyl transferase	1	1	2	1
Bilirubin	1	1	2	1
Triglyceride	7	2	4	1
Pre-albumin	At the start and after 5-7 days			
Albumin	Monthly	Monthly	1	1

Source: adapted from Nogueira RJ, Lima AE, Prado CC, Ribeiro AF. Nutrição em pediatria-oral, enteral e parenteral. 1a ed. Campinas: Sarvier; 2011. p. 318.^(72)^

* The frequency of monitoring can vary according to the clinical picture and the possibility of blood withdrawal.

As with other CF patients, when on prolonged PNT, they may require monitoring of other parameters, such as carnitine, taurine, selenium, copper, chromium, manganese, ferritin, B12, folic acid, vitamins A, D, and E, molybdenum, and ammonia^(72)^ (level of evidence 5).

## 4. Dietary counseling and behavioral intervention

### Is there a superior strategy for improving the quality of life of cystic fibrosis patients?

Yes. The behavioral intervention strategy associated with nutritional education has been shown to be more effective when compared to dietary counseling alone. Studies have revealed a significant increase in weight and stature gain and energy intake, when both strategies are associated with counseling for families of children with cystic fibrosis aged 1 to 12 years^(8,4,9,15,73-76)^ (level of evidence 2).

### Are there any validated instruments in Brazil for behavioral assessment of cystic fibrosis patients?

Yes. The Behavioral Pediatrics Feeding Assessment Scale (BPFAS) developed by psychologist William B. Crist was the most comprehensive and reliable instrument for the assessment of feeding behavior in pediatrics as evidenced by a systematic review conducted in 2015.^(77)^ It has been used in studies with patients with chronic disease and CF.^(78)^ In 2017 this questionnaire was validated in Brazilian Portuguese, so it can be applied in Brazilian CF patients^(79)^ (level of evidence 1).

### Is psychological intervention effective for nutritional treatment compliance?

Yes. Cognitive-behavioral therapy is the psychological intervention most indicated as a strategy for prevention and treatment of nutritional disorders in fibrocystic patients, with effective results in improving compliance to nutritional treatment.

The identification of inappropriate eating behaviors must be detected early and allow a joint intervention between the dietician and the psychologist in the approach towards the patient^(4,10,14,80,81)^ (level of evidence 2).

### What behavioral approaches to nutritional counseling can be used for cystic fibrosis patients?

The nutritional care plan should be individually tailored and incorporate behavioral strategies to be successful in meeting current nutritional guidelines for cystic fibrosis.^(8,15,79)^ Some of the intervention strategies include increasing dietary calories gradually, one meal at a time; recognizing, praising, and rewarding the child at each meal at which the caloric goal is met; offering mini-meals at snack times; teaching parents alternatives for dealing with slow eaters; limiting meal times to 15 minutes for young children; identifying appropriate rewards for the child eating the expected amount of food; and praising appropriate eating behaviors^(4,9,15,79,82,83)^ (level of evidence 5).

## 5. Special situations

### Is there a specific nutritional management of pregnancy and lactation in women with cystic fibrosis?

Yes. Additional energy requirements range from 200kcal to 300kcal per day from the beginning of gestation,^(84,85)^ but patients with intestinal malabsorption due to pancreatic insufficiency and sub-optimal BMI may require higher energy and protein intake.^(86)^

As for vitamin A, in a small group of pregnant women with CF, supplementation within the usual range of 18,000IU per day was compatible with normal serum levels.^(87,88)^ For safer prescriptions, it is important to assess intake and serum level in the preconception period and during pregnancy, because both severe deficiency and excess are teratogenic, and are associated with adverse reproductive conditions.^(89,90)^ Vitamin D should be supplemented, according to serum level, even in women with sufficient pancreatic function.^(84)^ The other vitamins and minerals follow the same recommendations for pregnant and nursing non-fibrocystic women (level of evidence 5).

### Should patients with cystic fibrosis-related diabetes restrict carbohydrates in their diet?

No. There is no indication for carbohydrate restriction for patients with CF and CF-related diabetes or glucose intolerance. It is recommended to choose carbohydrates with a low glycemic load for habitual consumption in these patients, and the routine intake of sucrose and foods with simple sugars should be discouraged, respecting the appropriate glycemic control of each patient. Carbohydrate counting strategy is a good tool for glycemic control, according to variations in carbohydrate intake, although it depends on the adequate understanding of the patient or caregivers^(91-95)^ (level of evidence 5).

### Should patients with cystic fibrosis-related hepatopathy restrict lipids in their diet?

No. The goal of nutritional support is to slow disease progression and to treat symptoms.^(96,97)^ There is no association between hepatic steatosis in CF and malnutrition, but there is a significant association with higher BMI.^(98)^ An adequate energy intake should be maintained in the diet, including correction of serum levels of fat-soluble vitamins^(96,99)^ (level of evidence 5).

### Are there dietary strategies for preventing osteopenia and osteoporosis specific to cystic fibrosis? Moreover, for patients who already have such a disease?

Yes. Osteoporosis and osteopenia are highly prevalent in CF patients due to a variety of factors, including direct effects of CFTR dysfunction on bone cells, pancreatic insufficiency, chronic inflammation, physical inactivity, and the use of some medications.^(100-104)^Nutritional strategies include optimization of lean body mass and BMI, close monitoring of vitamin D and K ( despite widespread supplementation, low biochemical levels have been observed in this population) and calcium supplementation, if needed.^(102,105,106)^Dietary protein balance is also important to prevent muscle loss^(28)^ (level of evidence 2).

### Is there a difference in dietary treatment for cow’s milk protein allergy, pancreatitis, and celiac disease in patients who also suffer from cystic fibrosis?

No. Investigation for other diseases should be performed in any CF patient with persistent gastrointestinal symptoms without improvement with standard treatment.^(107-111)^The clinical features of these diseases may be similar, despite the difference in pathogenesis.^(111,112)^Malabsorption symptoms, commonly present in CF, may delay the diagnosis of celiac disease.^(107,109)^ Daza et al., in a 4-year follow-up, diagnosed 14.8% patients (4/27) with food allergy,^(113)^ 2.13% (n=230) with celiac disease^(111)^ and 1.24% with pancreatitis.^(114)^ There is currently no scientific justification to restrict gluten, milk and dairy products in the diet of CF patients, unless diagnosed with these conditions (level of evidence 5).

### What specific dietary management should be performed in the diet of obese CF patients?

There are not enough studies for nutritional recommendations for obese CF patients. Thus, a balanced and diversified diet should be followed without restrictions, since there is worsening of lung function related to excess adiposity^(115,116)^ (level of evidence 5).

### Are there specific recommendations for the diet of cystic fibrosis patients when practicing sports?

No publications were found regarding specific nutritional recommendations for patients with active CF, and the same guidelines should be followed as for athletes in general.^(117,118)^ It is advisable to check if there is energy supply for increased demand and adequately replenishment of fluids and electrolytes^(119)^ (level of evidence 5).

### Is there a specific recommendation for the use of probiotics in cystic fibrosis?

There are not enough studies with good methodological quality for therapeutic recommendations with probiotics regarding strain, amount, and duration of treatment in CF,^(120,121)^ although the use of probiotics in CF has a promising future for reducing intestinal inflammation,^(122-125)^ decreasing respiratory exacerbations,^(126-129)^and improving quality of life.^(126,128,130,131)^

It is not advisable to use probiotics or symbiotics in patients undergoing lung transplantation because of the opportunity for bacterial translocation (immunosuppressed patients). In clinical practice, it is released for consumption only one year after transplantation and with medical/nutritional supervision^(132)^ (level of evidence 3).

### Is there specific nutritional therapy after lung transplantation in cystic fibrosis?

Before transplantation, nutritional care seeks the adequacy and/or maintenance of an adequate nutritional status (for adults, BMI between 17 and 27kg/m^2^, due to the lower mortality observed in this range up to 90 days after transplantation;^(133)^ for children and adolescents, a percentile above the 3^rd^ in the BMI/age curves is recommended).^(134,135)^However, a recent systematic review has not demonstrated association between BMI and post-transplantation mortality^(136)^ (level of evidence 2).

In the immediate postoperative period, there are no specific recommendations for CF patients undergoing lung transplantation. The oral diet is introduced on the first or second day, 6 hours after extubation. The evolution of the diet should be according to institutional protocol, according to the patient’s acceptance and tolerance, and with adequate enzyme replacement. Oral complementation must be started when food tolerance and acceptance are compromised. If the patient is not able to start the oral diet on the third or fourth postoperative day, or is dependent on noninvasive ventilation, enteral diet via tube or gastrostomy should be started. Parenteral nutrition is indicated in the presence of a non-functioning gastrointestinal tract, a situation that occurs depending on the surgical technique used.^(137,138)^

In the postoperative period of lung transplantation, constipation and distal obstruction syndrome are common, and the latter can evolve to sepsis. A fluid supply and a diet rich in fiber are recommended to help bowel movement, which should be reestablished in the first 48 to 72 hours after surgery. Otherwise, laxatives/fleet enema should be administered.^(139)^

The patient should avoid the consumption of grapefruit, as well as foods that contain it their composition, due to the interaction between drug and nutrient. Grapefruit is not commonly consumed by Brazilians, but it may be present in the composition of some foods, such as citrus juices and soft drinks. Grapefruit juice inhibits cytochrome p450, altering the metabolism of immunosuppressive medication, increasing its plasma concentration, and leading to the risk of overdose and adverse effects.^(140)^

In the late postoperative period, food intake usually increases, patients often gain weight, and the nutritional status is reestablished. From birth to 24 months, the p50 target for weight/height is suggested; from 2 to 20 years, the p50 target for BMI and, for older than 20 years, the BMI target of 23kg/m^2^ for men and 22kg/m^2^ for women. Some patients may become overweight if they are still on a hypercaloric and hyperprotein diet. Patients with a gastrostomy can reduce their use once their food intake increases and their nutritional status is restored. Ideally, the gastrostomy should be removed when a BMI>19kg/m^2^ is reached, with maintenance of this index without complementary nutrition for 3 to 6 months^(139)^ (level of evidence 5).

Tables 4 and 5 present the nutritional recommendations and the main changes and management in lung transplantation (Table 5).

**Table 4:** Nutritional recommendations in lung transplantation(137)

Nutrient	Immediate postoperative period	Late postoperative period
Calories	130%-150% of baseline energy expenditure	Sufficient for maintaining healthy weight
Proteins	1.5-2.0g/kg/day	0.8-0.9g/kg/day
Carbohydrates	50%-70% of non-protein calories	Avoid excessive food
Fats	30%-50% of the total calorie value	Monitor the concentration of cholesterol and triglycerides
Liquids	Restrict only if there is hyponatremia or edema	Restrict only if there is hyponatremia or edema
Electrolytes	Monitor levels of potassium, magnesium, phosphorus, and sodium. Supplement sodium if necessary	Monitor levels of potassium, magnesium, phosphorus, and sodium. Supplement sodium if necessary
Vitamins and minerals	Supplementation of vitamins and minerals as per the RDA; consider the post-transplant complications; monitor fat-soluble vitamins and supplement, if necessary; supplement calcium and vitamin D	Supplementation of vitamins and minerals as per the RDA; consider the post-transplant complications; monitor fat-soluble vitamins and supplement, if necessary; supplement calcium and vitamin D

RDA: Recommended Dietary Allowance.

**Table 5:** Main changes related to post-lung transplantations and their respective management(139,141)

Etiology	Medication	Treatment	Risk of non treatment
Obesity	Corticoids	Physical activity, healthy eating habits, and changes in lifestyle	Development of *diabetes mellitus*, hypertension, hyperlipidemia, and increased cardiovascular risk
*Diabetes mellitus*	Corticoids and cyclosporine	Insulin therapy	Poor healing, retinopathy, neuropathy, vascular disease, kidney disease, graft failure
Hypertension	Corticoids	Normal salt intake diet (5g salt/day) and antihypertensive medication	Vascular disease, kidney disease, and graft failure
Osteoporosis	Corticoids	Adequate consumption of calcium, supplementation of calcium and vitamin D, monitor bone density, parathyroid hormones, vitamin D metabolism, calcium, and levels of blood magnesium	Joint diseases, inactivity
Hyperlipidemia	Cyclosporine and corticoids	Diet with adequate saturated fat and cholesterol content, physical activity, maintenance of a healthy weight, and lipid-lowering medication	Peripheral vascular disease, coronary artery disease, and graft failure
Hyperkalemia	Cyclosporine and tacrolimus	Diet adequate in potassium	Cardiac arrhythmia

## 6. Enzyme replacement and gastrointestinal manifestations

### What are the guidelines for the correct use of pancreatic enzymes?

Pancreatic enzyme replacement therapy (PERT) is recommended for all patients who have evidence of pancreatic insufficiency,^(142)^ and are listed on table 6. In clinical practice, the administration of enzyme microspheres to infants can be difficult. If the infant refuses to take the enzyme microspheres on a spoon with some breast milk or formula, the administration with an acid apple puree, *e.g.,* may be successful. If the child still refuses the microspheres, the use of unprotected powdered enzymes may be temporarily considered. Pancreatic enzymes should never be added to the diet.^(4)^ When enteral nutrition is used, oral PERT should be maintained. If this is impossible, the enzymes should not be mixed with food; they should be given bolus via the enteral route. There is insufficient evidence to provide specific recommendations for the administration of PERT via an enteral feeding tube. There are different infusion times, and the need for enzyme administration may vary, *e.g.,* in a continuous or bolus fashion. Enzymes are usually given at the beginning and end of the infusion, but new devices for administering PERT are being developed. Bolus feeds may require a higher dose due to the increased rate of fat infusion. In patients taking PERT by mouth, who do not voluntarily wake up or do not wish to wake up during the night, it may be more practical to determine the total dose of PERT needed and give 50% of dose at the beginning of feeding and 50% at the end of feeding. Pancreatic enzymes should not be ground or mashed. When powdered or unprotected enzymes are used, the addition of a proton pump inhibitor may help prevent lipase destruction by gastric acid.^(4)^

**Table 6:** Pancreatic enzyme lipase replacement therapy: consensus guidelines(1,4,5,9)

Age	Supplementation
Infants (up to 12 months)	2,000-4,000U lipase/120mL of formula or estimated consumption of maternal milk and approximately 2,000U of lipase/g dietary fat in foods
Children aged 1 to 4 years	2,000-4,000U lipase/g of dietary fat, increasing the dose as is necessary (maximum dose of 10,000 lipaseU/kg per day)
Children aged >4 years and adults	Consider starting at 500U lipase/kg/meal, titrating up to a maximum dose of: - 1,000-2,500U lipase/kg/meal OR - 10,000U lipase/kg/day OR - 2,000-4,000U lipase/g of dietary fat obtained from all meals, snacks, and beverages containing fat

It is important to monitor growth and/or nutritional status at regular intervals to determine the need for PERT or adequacy of treatment at every clinic visit for infants, every 3 months for children and older adolescents, and every 6 months for adults^(4)^(level of evidence 1 for enzyme replacement; level of evidence 5 for monitoring of replacement).

### What is the prevalence and symptoms of gastroesophageal reflux in cystic fibrosis? Is there a differential treatment for gastroesophageal reflux disease in cystic fibrosis?

The prevalence of documented gastroesophageal reflux disease (GERD) in CF varies widely across studies due to differences in age and diagnostic methods. Studies using impedance, detected GERD in 67% in a pediatric group and in 87% in adults.^(143,144)^

The main symptoms are regurgitation, vomiting, and abdominal pain in younger children, and retrosternal pain, dysphagia, and heartburn in adolescents and adults. Many patients are asymptomatic.^(145)^

The treatment is similar in patients with and without CF, and consists of dietary and behavioral measures (reducing the volume and range of diets, avoiding excessive alcohol and caffeine intake), gastric acid inhibiting medications, such as H2 receptor antagonists and proton pump inhibitors, and, in some cases, surgery.

Treatment of GERD in patients who will undergo lung transplantation appears to reduce the risk of rejection and improve lung function^(145)^ (level of evidence 4).

### Is there any specific nutritional management for the treatment of constipated patients with cystic fibrosis?

No. However, patients should not be treated with diet therapy alone, but should be referred to a specialized medical team. The latest ESPGHAN consensus for CF patients includes in the diagnosis the presence of abdominal pain and/or distension and, decreased frequency of spontaneous bowel movements and/or, increased stool consistency for a few weeks to months, associated with the relief of these symptoms by the use of laxatives. For constipated individuals, dietary fiber intake and increased hydration are important to aid treatment, but for constipated CF patients, there is still no consensus in the literature^(146,147)^ (level of evidence 5).

### What is the nutritional management for patients with meconium ileus?

The meconium ileus can present in two forms:^(148-150)^ simple, if obstruction of the terminal ileum by meconium, with dilation of the small intestine by meconium, gas, and liquids; and complicated, if associated with complications, such as volvulus, necrosis, intestinal atresia, giant meconium pseudocyst, and intestinal perforation. When perforation occurs near delivery, meconium peritonitis occurs.^(149,150)^

Patients with complicated meconium ileus usually undergo a more extensive surgical intervention requiring prolonged fasting. In these cases, parenteral nutrition may be one of the modalities of nutritional therapy. In its composition, the use of structured lipids is suggested, with an association of medium chain triglycerides (MCT) and fish oil to minimize the risk of cholestasis.^(149,150)^

As soon as possible, the gastrointestinal tract should be used by the oral route or in the form of enteral nutritional therapy, starting with smaller volumes of diet and slow progression. In the absence of breast milk and in the presence of complicated conditions with necrosis, peritonitis, or intestinal resections, a formula with extensively hydrolyzed protein or an elemental diet can be used.^(150)^

In the presence of an enterostomy, there may be increased sodium and water loss, leading to metabolic acidosis, electrolyte disturbance, and difficulty in weight gain.^(151)^

Most children with CF and meconium ileus have pancreatic insufficiency, and confirmation can be made with fecal elastase. Feces should be collected from the rectum whether pasty or formed, and not from the enterostomy. Elastase in liquid stool can result in falsely low values. As soon as the diet is initiated, pancreatic enzyme should also be started^(150,152)^ (level of evidence 4).

## CONCLUSION

A scientific consensus with a practical format (questions and answers) on the nutritional management of cystic fibrosis was developed. It should be applied by professionals involved in nutritional therapy in reference centers for the treatment of cystic fibrosis in Brazil.
